# Human Herpesvirus 6A (HHV-6A) Encephalitis in an Immunocompetent Patient and Its Association With Glioblastoma: A Case Report

**DOI:** 10.7759/cureus.80780

**Published:** 2025-03-18

**Authors:** Rute Aleixo, Rosa Sá, Isabel Ramos, Cristina Valente

**Affiliations:** 1 Infectious Diseases, Unidade Local de Saúde de Coimbra, Coimbra, PRT

**Keywords:** glioblastoma, human herpesvirus 6, meningoencephalitis, neuro-oncology, viral oncogenesis

## Abstract

Human herpesvirus 6 (HHV-6) is a neurotropic virus capable of establishing latency in the central nervous system. While its reactivation is well-documented in immunocompromised individuals, its role in immunocompetent hosts remains unclear. Additionally, growing but inconclusive evidence suggests a potential association between HHV-6 and glioblastoma.

We present the case of a 65-year-old immunocompetent male who developed HHV-6A-associated meningoencephalitis, followed by the diagnosis of high-grade glioblastoma within months. The patient initially presented with altered consciousness, seizures, fever, and right-sided motor deficits, leading to a diagnosis of HHV-6A meningoencephalitis confirmed by cerebrospinal fluid and plasma polymerase chain reaction. Despite clinical improvement with antiviral therapy, he developed progressive neurological symptoms two months later, and neuroimaging revealed multiple expansile lesions with significant mass effects. A stereotactic brain biopsy confirmed the diagnosis of glioblastoma, isocitrate dehydrogenase-wildtype, and the patient ultimately succumbed to the disease.

The temporal association between HHV-6A infection and glioblastoma raises critical questions about its potential role in tumorigenesis. While previous studies have detected HHV-6 DNA and proteins in glioma tissues, supporting hypotheses of viral-mediated inflammation, immune modulation, and oncogenic interactions, a direct causal link remains unproven. Additionally, the patient’s treatment with valganciclovir, an antiviral explored as a potential glioblastoma adjuvant therapy, prompts discussion about its possible influence on tumor progression. Further research into HHV-6’s oncogenic potential may provide valuable insights into gliomagenesis and open avenues for novel therapeutic strategies, including antiviral approaches in glioblastoma management.

## Introduction

Human herpesvirus 6 (HHV-6) is a linear, double-stranded DNA virus belonging to the Herpesviridae family, specifically the Betaherpesvirinae subfamily, which also includes cytomegalovirus (CMV) and human herpesvirus 7 (HHV-7) [1,2]. HHV-6 exists as two distinct variants, HHV-6A and HHV-6B, which differ in epidemiology, cellular tropism, and pathogenicity. HHV-6B is highly prevalent, infecting over 90% of the population within the first three years of life, and is the primary cause of roseola infantum, commonly associated with febrile seizures and encephalitis in children [2]. In contrast, HHV-6A is less well-characterized but has been suggested to have greater neurotropism and potential oncogenic properties, particularly in the central nervous system (CNS) [1,2]. Unlike HHV-6B, which primarily affects children, HHV-6A is more frequently detected in adult neurological disorders, including multiple sclerosis and encephalitis, and has been implicated in glioma pathogenesis [1,2]. Both variants establish latency in various tissues, including the CNS, and their reactivation can lead to significant neurological manifestations such as meningoencephalitis [3]. Beyond neurological conditions, HHV-6 has been identified in various tumors, including glioma, oral cancer, cervical cancer, adrenocortical tumors, gastrointestinal cancer, classical Hodgkin lymphoma, and non-Hodgkin lymphoma [4]. Despite these associations, the role of HHV-6, particularly HHV-6A, in gliomagenesis remains unclear. While some studies have reported the presence of HHV-6 DNA and proteins in glioma tissues, the significance of these findings remains debated, and no direct oncogenic role has been confirmed. Further research is needed to better understand the potential contribution of HHV-6A to glioblastoma pathogenesis [4].

Glioblastoma is the most aggressive primary malignant brain tumor in adults, characterized by rapid progression and poor prognosis. Although the exact etiology of glioblastoma remains unclear, viral infections, including HHV-6, have been suggested as potential contributors to gliomagenesis [3,5]. Several studies have detected HHV-6 DNA in glioma tissues, raising the question of whether it plays an active role in tumor development [5,6]. However, the relationship between HHV-6 and glioblastoma remains controversial, with limited and inconclusive evidence supporting a direct causative role [7]. Further research is needed to determine whether HHV-6A plays an active role in glioblastoma pathogenesis or is merely incidental.

In this case report, we present a 65-year-old immunocompetent male who initially developed HHV-6A-associated meningoencephalitis, followed by a diagnosis of high-grade glioblastoma within months. Given the close temporal relationship between these two conditions, this case allows one to explore the potential interplay between HHV-6A infection and CNS malignancies. By presenting this case, we aim to contribute to the ongoing discussion of HHV-6A’s oncogenic potential and highlight the need for further research into its role in glioblastoma development.

## Case presentation

A 65-year-old autonomous male patient with a history of hypertension and dyslipidemia presented to the emergency department on March 13, 2022, with altered consciousness, psychomotor agitation, fever, and right-sided motor deficits. According to family members, he had experienced general malaise, tremors, and nausea the night before. The following morning, he was found collapsed, exhibiting a vacant stare, tremors in the left limbs, and sialorrhea lasting less than a minute. He had two similar episodes without regaining consciousness between them, and emergency medical responders observed another seizure before hospital arrival.

On admission, he was febrile (38°C) and presented with right-sided hemiparesis and an altered level of consciousness, with a Glasgow Coma Scale (GCS) score of 8 (eye opening 4, verbal response 1, and motor response 3). Intermittent myoclonic movements were noted, leading to the initiation of levetiracetam infusion, which improved motor function but did not restore consciousness. Initial laboratory tests revealed leukocytosis (14,590/µL) and an elevated C-reactive protein of 1.7 mg/dL. A cranial CT scan performed the same day showed no alterations. A lumbar puncture revealed an elevated protein concentration of 65.9 mg/dL with no pleocytosis or other abnormalities. A multiplex polymerase chain reaction (PCR) panel for infectious meningoencephalitis pathogens was performed, testing for *Herpes simplex virus type 1*, *Herpes simplex virus type 2*, enteroviruses, *Varicella-zoster virus*, HHV-6, *Human parechovirus*, CMV, and several bacterial pathogens, including *Neisseria meningitidis*, *Streptococcus pneumoniae*, *Haemophilus influenzae*, *Escherichia coli *K1, *Streptococcus agalactiae*, *Listeria monocytogenes*, and *Cryptococcus neoformans/gattii*. The test detected HHV-6 DNA in the cerebrospinal fluid (CSF).

With suspicion of viral meningoencephalitis, he was admitted to the infectious diseases department with a GCS score of 11 and started on intravenous ganciclovir (320 mg every 12 hours). The ganciclovir dose was calculated based on a regimen of 5 mg/kg, adjusted to renal function. To minimize nephrotoxicity risk, adequate hydration was ensured, guaranteeing sufficient fluid intake and electrolyte balance.

**Figure 1:**
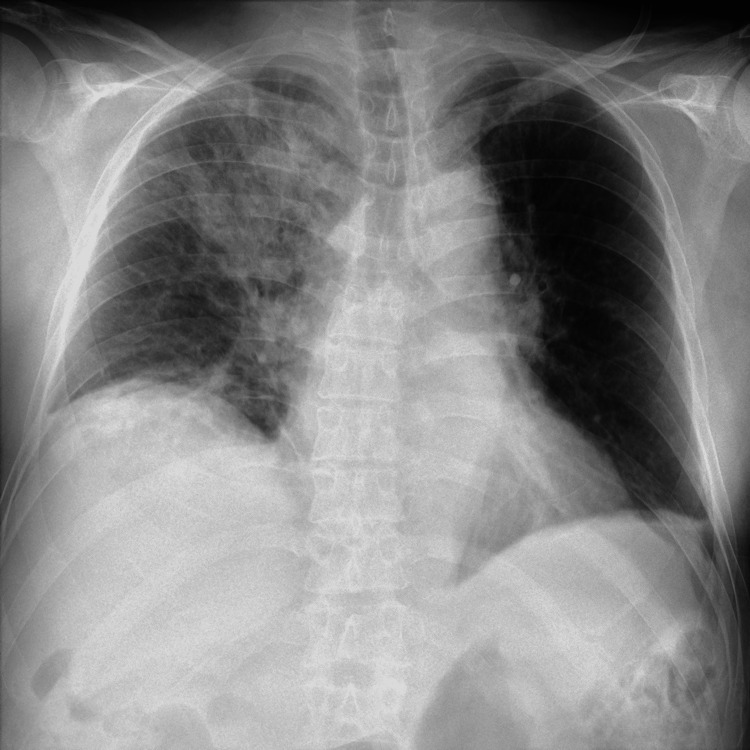
Chest X-ray showing an infiltrate in the right lung involving the perihilar region and upper lobe

The patient’s neurological status remained altered, with progressive respiratory deterioration, leading to the initiation of dexamethasone (10 mg every eight hours). A chest X-ray performed on March 15 revealed an infiltrate in the right lung involving the perihilar region and upper lobe (Figure 1). On the same day, he was transferred to the intensive care unit due to respiratory failure, requiring mechanical ventilation and vasopressor support. On March 16, bronchoalveolar lavage was performed, revealing the presence of HHV-6A DNA by PCR, with no other pathogens or microorganisms identified. Simultaneously, a repeat lumbar puncture was performed for CSF HHV-6A DNA quantification and plasma HHV-6A viral quantification. Both revealed high viral loads. These findings established a diagnosis of HHV-6A systemic viral dissemination with pneumonitis and CNS involvement.

After five days of mechanical ventilation, he was extubated on March 19 and remained hemodynamically stable. He was transferred back to the infectious diseases department on March 20. Electroencephalography on March 15 demonstrated diffuse slowing with predominant left temporal dysfunction and epileptiform discharges, while a repeat study on March 30 showed persistent left temporal slowing without epileptiform activity.

**Figure 2:**
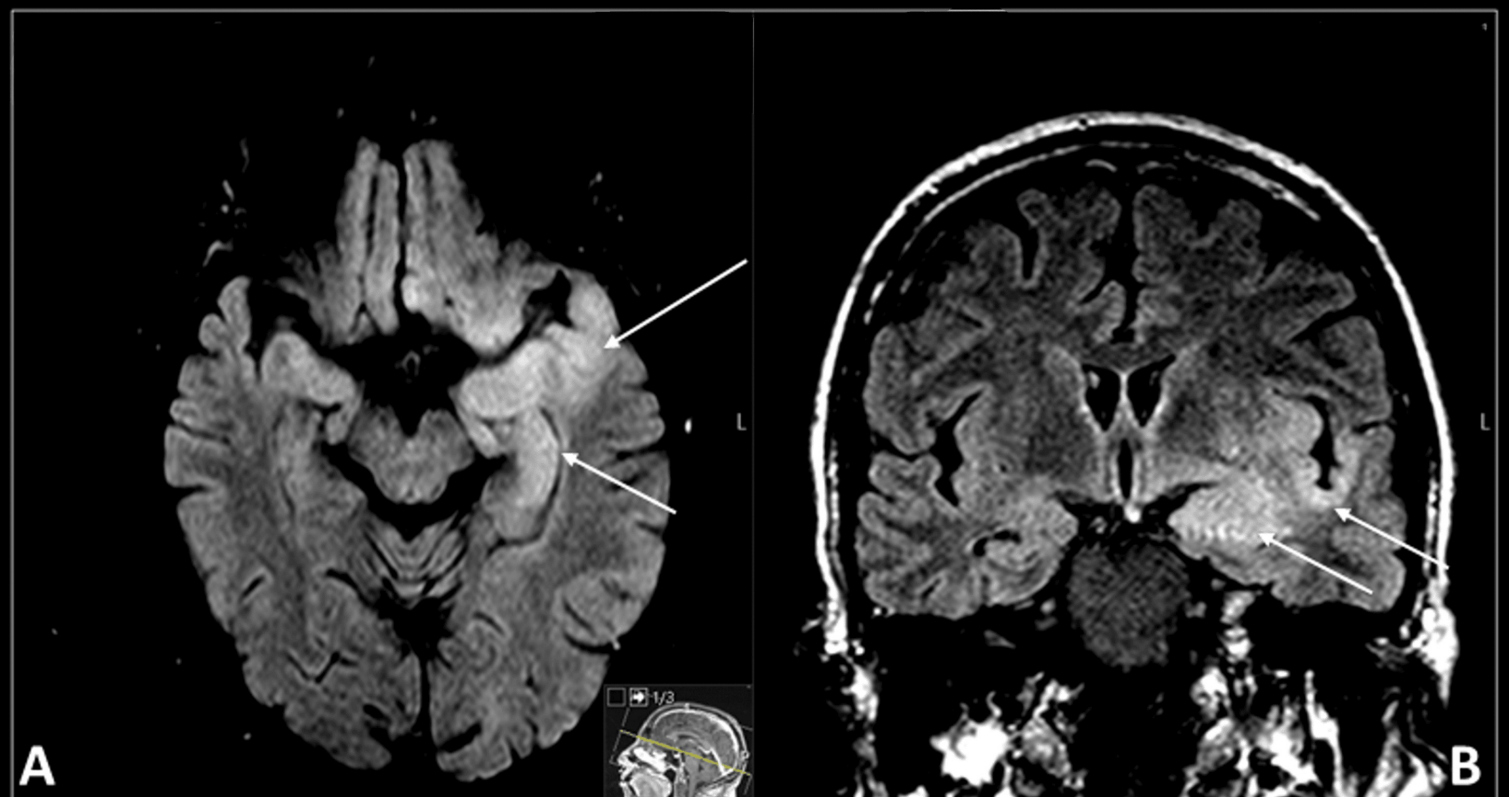
Brain MRI showing parenchymal hyperintensity predominantly visible on FLAIR sequences, consistent with limbic encephalitis. (A) Axial FLAIR image highlighting hyperintensity in the left hippocampus and amygdala (arrows), with mild cortical swelling. (B) Coronal FLAIR image demonstrating hyperintensity in the left insular cortex and anterior temporal cortex (arrows) MRI: magnetic resonance imaging, FLAIR: fluid-attenuated inversion recovery

A brain MRI performed on March 22 showed significant signal alterations, characterized by parenchymal hyperintensity predominantly visible on fluid-attenuated inversion recovery (FLAIR) sequences, involving the left hippocampus, amygdala, insular cortex, and anterior temporal cortex, as well as a focal area of lenticular hyperintensity on the same side. A mildly swollen cortical pattern was observed, along with areas of diffusion restriction on diffusion-weighted imaging, primarily affecting the amygdala and hippocampus. These findings suggested the hypothesis of limbic encephalitis in an inflammatory process secondary to herpesvirus infection (Figure 2). A follow-up MRI on April 5 showed no significant changes compared to the previous study.

Serial quantitative PCR showed a progressive decrease in viral load, with CSF HHV-6 DNA levels declining from 7,186 copies/mL on March 16 to 1,484 copies/mL on March 29, while plasma viral load decreased from 37,051 copies/mL on March 16 to 4,934 copies/mL on April 19. Throughout hospitalization, CSF cultures for mycobacteria, bacteria, and fungi were consistently negative, as were sequential blood and urine cultures. Additionally, subsequent lumbar punctures demonstrated a progressive decrease in protein concentration, ultimately normalizing.

No underlying immunosuppressive condition was identified. Human immunodeficiency virus, viral hepatitis, and tuberculosis screenings were negative, autoimmune encephalitis panels in CSF and serum were unremarkable, and lymphocyte subset analysis, immunoglobulin levels, and complement assessments were within normal ranges.

Dexamethasone tapering was completed by April 8, and the patient completed 37 days of intravenous ganciclovir followed by three days of oral valganciclovir 900 mg every 24 hours. Given his significant clinical and virological improvement, further lumbar punctures were deemed unnecessary. He was discharged neurologically intact on April 24, 2022, with a GCS score of 15, no focal neurological deficits, and a stable clinical condition. There were no signs of worsening or recurrence of symptoms, and he was considered fit for discharge with continued valganciclovir therapy and scheduled follow-up.

However, at the follow-up visit on May 11, he reported fatigue, anxiety, and mild cognitive complaints, while his wife described episodic confusion, right-hand tremors, and perception of discrete handwriting changes.

**Figure 3:**
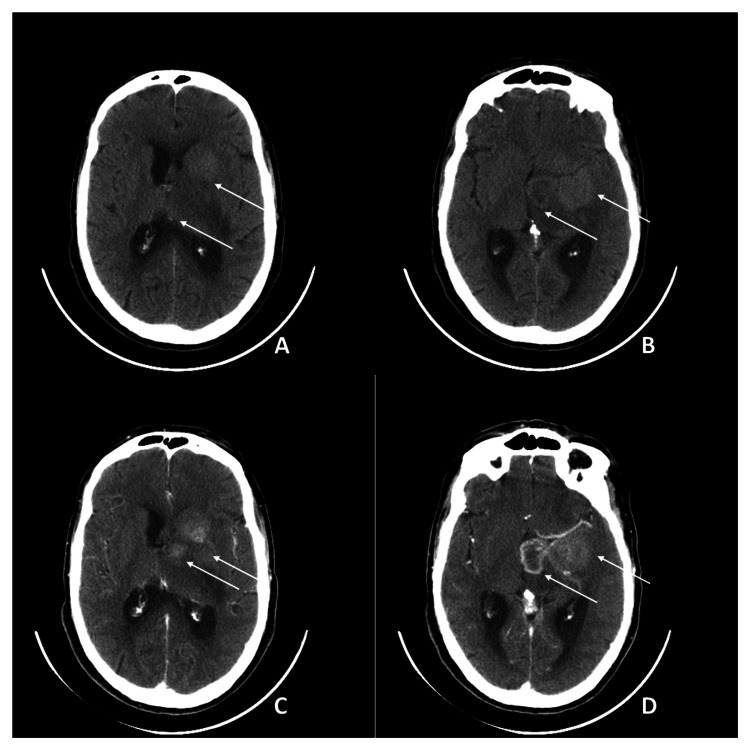
Brain CT scan revealing a new left hemispheric expansile lesion with pronounced edema, hydrocephalus, and midline shift. (A) Axial view highlighting the left medial temporal lesion (arrows), showing hyperdense areas and moderate heterogeneous enhancement. (B) Axial view focusing on the anterior thalamo-capsular and subthalamic lesions (arrows) with irregular ring enhancement and a likely necrotic core. (C) Axial view demonstrating significant edema extending inferiorly to the left mesencephalon and pons (arrows) contributing to mass effect and midline shift. (D) Axial view depicting the left anterior nucleo-capsular lesion (arrows) showing heterogeneous enhancement and a likely necrotic central area in association with hydrocephalus and midline shift CT: computed tomography

**Figure 4:**
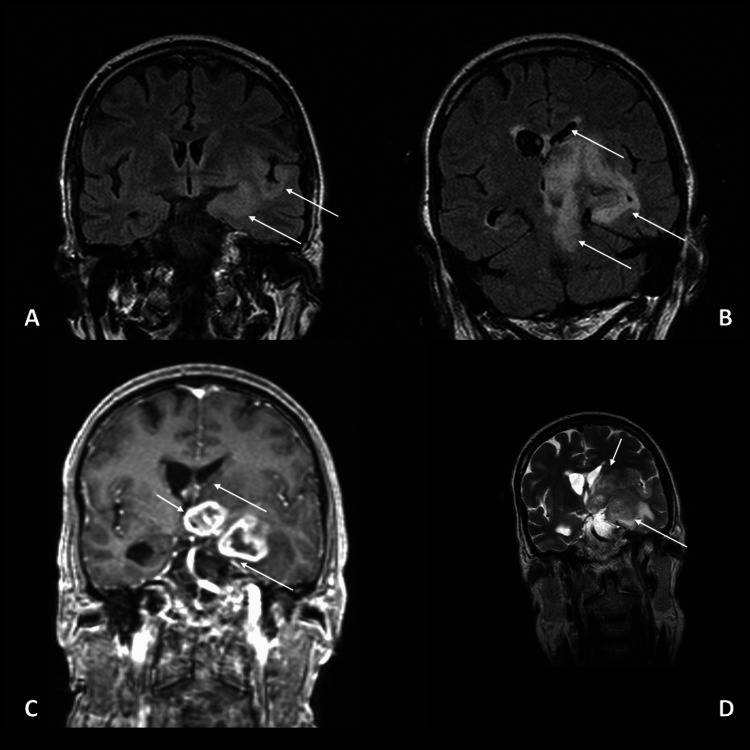
Brain MRI (coronal views) demonstrating multiple expansile lesions with heterogeneous enhancement, necrotic areas, significant edema, and mass effect. Arrows indicate the main areas of alteration. (A) Coronal view showing heterogeneous enhancement and surrounding edema in the amygdala, lenticulo-capsular, and anterior thalamo-capsular regions (arrows). (B) Coronal view revealing a poorly defined area of signal alteration with significant edema (arrows) contributing to mass effect and compression of adjacent structures. (C) Coronal view demonstrating two distinct lesions with irregular enhancement (arrows) located in the left thalamo-capsular and subthalamic regions with surrounding edema and compression of the left lateral ventricle. (D) Coronal view illustrating ventricular compression and midline shift (arrows) with evidence of hydrocephalus and ependymal edema MRI: magnetic resonance imaging

On May 15, he was readmitted with worsening gait imbalance and tremors in the right upper limb. A cranial CT scan performed the following day showed significant radiological worsening, with newly defined lesions of apparently expansive nature in the left medial temporal region, anterior thalamo-capsular and subthalamic areas, and left anterior nucleo-capsular region. These lesions were in close proximity to each other but maintained distinguishable contours. The left thalamic/subthalamic lesion appeared centrally hypodense, with irregular ring enhancement and a non-enhancing central area, likely necrotic. The left medial temporal and anterior nucleo-capsular lesions exhibited higher density, with hyperdense areas relative to the parenchyma, and showed moderate, heterogeneous enhancement. A central non-enhancing area, likely necrotic, was also observed in the medial temporal lesion. Moderate to marked edema was associated, extending inferiorly to the left mesencephalon and pons. A significant increase in mass effect was noted, causing deformation of the left lateral ventricle and the third ventricle, along with a slight rightward midline shift. Ventricular enlargement was present, with mild hydrocephalic dilation of the lateral ventricles. Given the imaging evolution, a neoplastic etiology was considered (Figure 3). After evaluation by the neurology and neurosurgery teams, he was admitted to the neurosurgery department, where valganciclovir was discontinued. A subsequent brain MRI on May 20 confirmed multiple expansile, rounded lesions in the left medial temporal lobe, anterior thalamo-capsular and subthalamic regions, and left lenticulo-capsular region. Significant surrounding edema, along with the lesions, contributed to a mass effect on adjacent structures. This resulted in compression of the left lateral ventricle, displacement, and compression of the third ventricle toward the right, as well as compression of the left mesencephalic region. Additionally, ventricular volume was increased, with emerging signs of developing hydrocephalus and ependymal edema. These findings strongly suggested a neoplastic lesion (Figure 4).

A stereotactic brain biopsy performed on May 24 confirmed glioblastoma, isocitrate dehydrogenase-wildtype. The patient was treated with dexamethasone, initially at 4 mg once daily, until the day of the biopsy. Following the procedure, the dosage was increased to 5 mg intravenously every eight hours for three days and then transitioned to 4 mg orally every eight hours for an additional three days. Upon discharge on May 30, the neurosurgery team recommended a tapering regimen.

**Figure 5:**
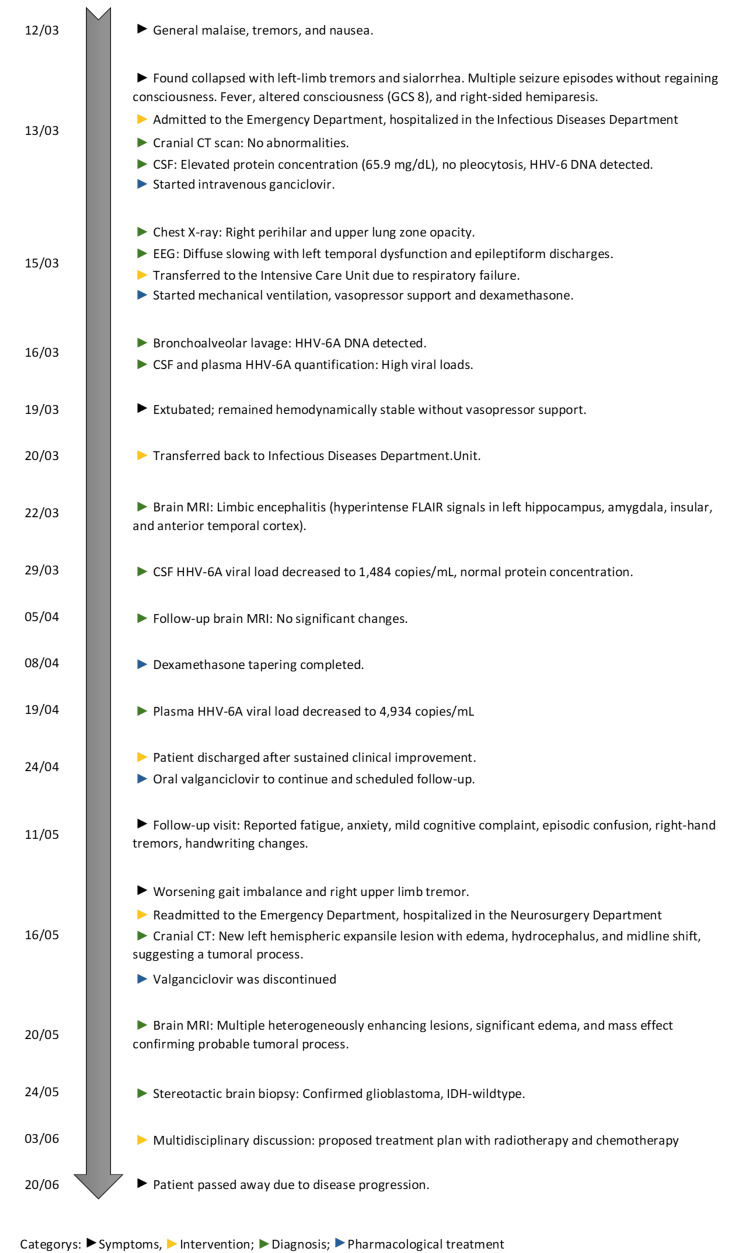
Timeline of the case report GCS: Glasgow Coma Scale, CSF: cerebrospinal fluid, CT: computed tomography, EEG: electroencephalogram, HHV-6A: human herpesvirus 6A, MRI: magnetic resonance imaging, FLAIR: fluid-attenuated inversion recovery, IDH: isocitrate dehydrogenase, DNA: deoxyribonucleic acid

Following a multidisciplinary discussion involving the neurosurgery and oncology teams on June 3, a treatment plan was proposed, including radiotherapy and chemotherapy. However, due to the patient’s clinical deterioration, the initiation of treatment was postponed, and the best supportive care was offered. Given the aggressive nature of the disease, he ultimately succumbed on June 20, 2022. For a more comprehensive understanding of the case’s chronological evolution, a timeline is presented in Figure 5.

## Discussion

HHV-6 is a neurotropic virus capable of establishing latency in the CNS. While HHV-6 reactivation is well-documented in immunocompromised individuals, its role in immunocompetent hosts remains an area of ongoing investigation. Although rare, cases of HHV-6 encephalitis in immunocompetent individuals have been reported, highlighting the importance of considering HHV-6 as a potential etiologic agent in encephalitic syndromes, even in the absence of immunosuppression [1,7-9].

In this case, an initially healthy 65-year-old male developed HHV-6 meningoencephalitis with a disseminated viral infection, confirmed by high HHV-6A DNA loads in CSF and plasma. The patient responded favorably to antiviral therapy, which decreased viral loads and showed clinical improvement. Although no approved therapies are specifically for HHV-6 infection, cidofovir, ganciclovir, and foscarnet have been utilized in treating HHV-6 disease, with noted clinical benefit [9]. However, two months later, he presented with focal neurological deficits, and imaging revealed space-occupying CNS lesions with mass effect, ultimately diagnosed as high-grade glioblastoma. In this case, the temporal association raises whether HHV-6A infection could play a role in glioblastoma development. However, it remains possible that the tumor was already present prior to the infection. However, the clinical and imaging findings strongly suggest the infection preceded the tumor diagnosis.

Several studies have detected HHV-6 DNA and proteins in glioma tissues, suggesting that HHV-6 might play a role in gliomagenesis. The proposed mechanisms include viral-mediated inflammation, immune evasion, modulation of cellular pathways involved in oncogenesis, and HHV-6-specific mechanisms [2,3,5,10-2]. In vitro studies indicate that HHV-6 proteins may contribute to glioma progression by altering the tumor microenvironment and promoting extracellular matrix degradation [4]. Additionally, HHV-6 has been implicated in the pathogenesis of other malignancies, suggesting a broader oncogenic potential [2,10-12]. However, whether HHV-6 is a direct contributor to glioblastoma formation or a coincidental finding in these tumors remains unclear.

Valganciclovir, an antiviral with activity against betaherpesviruses, has been investigated in glioblastoma treatment, particularly in CMV-positive tumors [13]. Some studies suggest that CMV infection in glioblastoma may contribute to tumor progression by modulating the immune microenvironment and promoting oncogenic signaling pathways [13,14]. The potential benefit of valganciclovir in glioblastoma has been explored in small clinical studies, with some reports indicating improved survival in patients receiving antiviral therapy [2,6,13]. However, these findings remain controversial, with limited large-scale evidence to support its routine use [4,13]. In this case, valganciclovir was discontinued upon the patient’s admission to the neurosurgery department prior to the glioblastoma diagnosis. Given the increasing evidence that HHV-6, like CMV, may play a role in gliomagenesis, the question of whether continued antiviral therapy could have impacted tumor progression arises.

While there is no direct evidence linking valganciclovir to glioblastoma suppression in HHV-6-associated cases, further research into antiviral strategies for herpesvirus-associated gliomas is warranted. This highlights whether antiviral therapy could influence glioblastoma growth and whether viral suppression alters tumor behavior.

Given the growing evidence implicating herpesviruses in gliomagenesis and other viruses [12], further research is essential to elucidate the mechanisms through which HHV-6 may contribute to tumor development. Molecular and epidemiological studies are needed to better define its role in glioblastoma and explore the potential therapeutic implications of antiviral treatment in its management.

## Conclusions

This case highlights a potential link between HHV-6A infection and glioblastoma, raising important questions about the role of viral infections in gliomagenesis. While HHV-6 has been detected in gliomas, its direct oncogenic role remains uncertain. Notably, some studies propose that antiviral agents could have therapeutic potential, particularly in glioblastoma cases associated with herpesviruses.

Further research is essential to better understand how viruses contribute to glioblastoma pathogenesis and assess whether antiviral strategies could influence disease progression. Clarifying the viral role in gliomagenesis may provide new therapeutic avenues, including antiviral interventions for glioblastoma treatment.
